# Resource competition in plant invasions: emerging patterns and research needs

**DOI:** 10.3389/fpls.2014.00501

**Published:** 2014-09-29

**Authors:** Margherita Gioria, Bruce A. Osborne

**Affiliations:** ^1^Department of Invasion Ecology, Institute of Botany, Academy of Sciences of the Czech RepublicPrùhonice, Czech Republic; ^2^University College Dublin School of Biology and Environmental Science, University College DublinDublin, Ireland; ^3^University College Dublin Earth Institute, University College DublinDublin, Ireland

**Keywords:** evolutionary adaptation, apparent competition, dominance, competitive ability, phenology, phenotypic plasticity, phylogenetic relatedness, resource gradient

## Abstract

Invasions by alien plants provide a unique opportunity to examine competitive interactions among plants. While resource competition has long been regarded as a major mechanism responsible for successful invasions, given a well-known capacity for many invaders to become dominant and reduce plant diversity in the invaded communities, few studies have measured resource competition directly or have assessed its importance relative to that of other mechanisms, at different stages of an invasion process. Here, we review evidence comparing the competitive ability of invasive species vs. that of co-occurring native plants, along a range of environmental gradients, showing that many invasive species have a superior competitive ability over native species, although invasive congeners are not necessarily competitively superior over native congeners, nor are alien dominants are better competitors than native dominants. We discuss how the outcomes of competition depend on a number of factors, such as the heterogeneous distribution of resources, the stage of the invasion process, as well as phenotypic plasticity and evolutionary adaptation, which may result in increased or decreased competitive ability in both invasive and native species. Competitive advantages of invasive species over natives are often transient and only important at the early stages of an invasion process. It remains unclear how important resource competition is relative to other mechanisms (competition avoidance via phenological differences, niche differentiation in space associated with phylogenetic distance, recruitment and dispersal limitation, indirect competition, and allelopathy). Finally, we identify the conceptual and methodological issues characterizing competition studies in plant invasions, and we discuss future research needs, including examination of resource competition dynamics and the impact of global environmental change on competitive interactions between invasive and native species.

## Introduction

Invasions by alien plants represent a major component of global change (Vitousek et al., 1996). Successful invasions occur when an alien species is capable of forming self-sustaining populations (naturalization) that may extend at considerable distances away from the original source of introduction, overcoming a range of biotic and abiotic barriers, along an introduction-naturalization-invasion continuum (Richardson et al., 2000; Richardson and Pyšek, 2012). Invasive alien species are known to alter the composition and diversity of the aboveground (e.g., Levine et al., 2003; Gaertner et al., 2009; Hejda et al., 2009) and belowground flora (Gioria and Osborne, 2010; Gioria et al., 2014) of many recipient communities, as well as impacting on a range of abiotic and biotic conditions, with potentially significant changes in the functioning of, and the services provided by, invaded ecosystems (see Ehrenfeld, 2010; Simberloff, 2011; Vilà et al., 2011; Eviner et al., 2012; Pyšek et al., 2012).

Resource competition, also known as exploitative competition, is a key process regulating plant community dynamics (e.g., Grime, 1973, 1977; Newman, 1973; Harper, 1977; Tilman, 1982, 1988) and has long been considered as a major mechanism determining the success of several invasive species (Elton, 1958; Tilman, 1997; Levine et al., 2003; Vilà and Weiner, 2004). In particular, a capacity for many invasive species to reduce diversity and to form nearly mono-specific stands (e.g., Beerling et al., 1994; Tiley et al., 1996; Gaertner et al., 2009) has often been attributed to a superior capacity of invasive species to compete for resources (Levine et al., 2003; Vilà et al., 2003; Vilà and Weiner, 2004) and/or due to the disproportionately greater effects of increases in resources on the performance of invasive vs. native species (e.g., Daehler, 2003; Leishman and Thomson, 2005; Funk, 2013).

In this paper, we review the literature on the role of resource competition in plant invasions. Specifically, we review studies comparing the competitive ability of invasive species vs. that of co-occurring native species, along a range of environmental gradients, distinguishing between the importance of competitive interactions below- and above-ground as well as that of intra- vs. interspecific competition; we report on the findings of studies accounting for phylogenetic relatedness through comparisons of invasive and non-invasive congeners, and of those comparing the competitive ability of dominant aliens vs. dominant natives; we discuss how the outcomes of resource competition may depend on other mechanisms, including phenotypic plasticity as well as evolutionary adaptation that may lead to increased or decreased competitive ability in both invasive and native species, dispersal and recruitment limitation, and competition avoidance that may result from phenological differences or from niche differentiation in space; we report on how indirect competition and allelopathy may interact with resource competition and on how a superior competitive ability of invasive species over that of native species may be only transient and change over time. Finally, we identify the conceptual and methodological issues characterizing research needs in this field and we discuss future research directions, including examinations of the potential impact of global environmental changes on resource competition between invasive and native species.

## Competitive ability in invasive species

### Defining resource competition, competitive ability and dominance

Two major questions in the field of invasion ecology relate to the competitive ability of invasive species and how this is affected by resource availability: (1) are invasive species superior competitors over native species; and (2) how do competitive interactions for resources between invasive and native species vary (over time) along environmental gradients? (see Glossary).

Resource competition is a negative interaction between individuals associated with a requirement for shared limiting resources (light, nutrients, and water) resulting in a reduction in one or more fitness components at the individual or at the population level (see Glossary; Goldberg et al., 1999). From a functional point of view, competition can be regarded as an alteration of the processes of (1) “acquisition” of resources, (2) their “allocation” to different parts, and contribution to overall plant performance and (3) the “deployment” of these parts in space (Bazzaz, 1996), by neighboring individuals.

Based on the above definitions, the competitive ability of a species can be broadly regarded as the ability of a species to acquire and/or make the best use of limiting resources, and/or a capacity to cope with low resource levels or to reduce the availability of resources to its neighbors. Such an ability is dependent on a combination of species traits that allow a species to compete for resources with neighboring individuals or species (see Weiner, 1993), including relative growth rate, height, lateral spread, storage organs, shoot thrust, leaf and root longevity, leaf nutrient concentration, specific leaf area, active foraging capability, response to damage, and palatability (Grime, 1998). Individual plants, however, vary greatly in their tolerance to different levels of available resources, making the concept of competitive ability at the species level strongly context-dependent (Tilman, 1982, 1988; see Weiner, 1993).

Goldberg (1990) pointed out that the competitive ability of a species can be classified into two components, as each individual has an effect on and responds to its environment, including its neighbors: (1) a “competitive effect”, which can be regarded as the ability of an individual to take up resources (high rates of resource acquisition), ultimately resulting in a reduction in the resources available to neighboring plants; and (2) a “competitive response”, i.e., the response of a species to reduced resource levels by competitors, which depends on a species' ability to tolerate low resource conditions associated with the presence of neighbors. These mechanisms, which can also be summarized into maximum resource capture vs. minimum resource requirements are not mutually exclusive (Suding et al., 2004).

Invasive species may achieve dominance via an innately superior competitive ability over that of native species arising from physiological advantages that include high rates of resource acquisition (e.g., Eliason and Allen, 1997; Alpert et al., 2000; Callaway and Aschehoug, 2000; Rejmánek, 2000; Pyšek and Richardson, 2007; van Kleunen et al., 2010, 2011; Matzek, 2012; Funk, 2013), such as a capacity to fix nitrogen (e.g., Atwood et al., 2010; Le Maitre et al., 2011; Gioria and Osborne, 2013) or an ability to tolerate low resource availability (see Tilman, 1982; Weiner, 1993; Goldberg, 1996; Craine et al., 2005; Funk, 2013). Dominance can also be achieved via mechanisms that may interact with resource competition and that will be discussed throughout this paper, including: (1) competitive advantages arising from the release from natural enemies that are present in their native range but not in the invasive range (Enemy Release Hypothesis; ERH; Keane and Crawley, 2002; Mitchell and Power, 2003; Callaway et al., 2004; Maron et al., 2014); (2) an increased competitive ability post-introduction arising from evolutionary changes leading to a reallocation of resources from defense mechanisms that may be required in their native range, to growth and development (Evolution of Increased Competitive Ability; EICA; Blossey and Nötzold, 1995); (3) high phenotypic plasticity in traits that allow the survival and spread in areas characterized by novel conditions (Bossdorf et al., 2005; Richardson and Pyšek, 2006; Davidson et al., 2011); (4) trait differences between alien and native species arising from phylogenetic distinctiveness (Mack, 1996; Rejmánek, 1996) and potentially reflecting differences in the ecological niches that can be occupied. This is based on early observations suggesting that competition with native species would favor the establishment of taxonomic distinct alien species (Darwin's Naturalization Hypothesis; Darwin, 1859); (5) pre-existing or acquired (via phenotypic and/or evolutionary responses) phenological differences that allow alien species that emerge earlier or persist longer to avoid resource competition in the early/later stages of development (Weiner, 1993; Wolkovich et al., 2013); (6) an ability to alter the abiotic (Ehrenfeld, 2010; Vilà et al., 2011; Pyšek et al., 2012) and biotic conditions in the invaded communities (White et al., 2006; Kulmatiski et al., 2008); (7) the release of allelochemicals that are potentially toxic to native species (Callaway and Aschehoug, 2000).

While the ERH predicts that the release from enemies confer an immediate competitive advantage to alien species, according to EICA, the invasive potential of alien species depends, at least in part, on their ability to evolve to reallocate resources previously destined to defense from natural enemies, thus they do not need to be competitively superior over native species at the time of introduction into a community. Evidence for ERH, EICA, and the Darwin's Naturalization Hypothesis is strongly context-dependent (e.g., Daehler, 2001; Duncan and Williams, 2002; van Kleunen and Schmid, 2003; Hierro et al., 2004; Bossdorf et al., 2005; Schaefer et al., 2011).

### Are invasive species superior competitors over native species?

Resource competition may play an important role in both the establishment (naturalization phase) and the spread (invasion phase) of invasive alien species, as well as in determining the magnitude and direction of the impact of plant invasions on invaded communities. Competitive advantages associated with a superior capacity to acquire resources have been regarded as a key factor responsible for the dominance of many alien species (*sensu* Grime, 1998) in the invaded communities (e.g., Tilman, 1997; Levine, 2000; Shea and Chesson, 2002; Levine et al., 2003; Seabloom et al., 2003; Stachowicz and Tilman, 2005).

Whether or not invasive plants are competitively superior over co-occurring native species thus represents a central question in invasion ecology. The competitive ability of invasive species has been compared to that of native species in several experimental studies in which co-occurring invasive and native species were grown separately or in mixtures, along a range of environmental conditions (see Vilà and Weiner, 2004; White et al., 2006 for reviews). Several studies have shown that many invasive species possess higher values of competitively advantageous traits than native and non-invasive species, including a superior capacity to acquire and retain resources and/or to advantageously exploit resources better than co-occurring native species (e.g., Huenneke et al., 1990; Burke and Grime, 1996; Rejmánek, 1996; Callaway and Aschehoug, 2000; Daehler, 2003; Leishman and Thomson, 2005; van Kleunen et al., 2010; Matzek, 2012), even in ecosystems with low-resource availability (Tecco et al., 2010; Funk, 2013). Also, there is evidence that resource competition does not necessarily play an important role in determining dominance by invasive plants (e.g., Mangla et al., 2011a) and a superior competitive ability is not a necessary condition for successful invasions (e.g., Corbin and D'Antonio, 2004; McGlone et al., 2012).

Conceptual and methodological issues have characterized many competition studies in invasion ecology, potentially affecting our understanding of the role of resource competition in plant invasions. First, the majority of studies have characterized the competitive ability of co-occurring invasive and native species indirectly, mainly by testing differences in biomass or other measures of plant growth or fitness, thus focusing on the *outcomes* of competition and between invasive and native species, rather than the *process* of competition (see Trinder et al., 2013 for a discussion between direct and indirect measurements of plant competition). Another potentially significant issue is associated with the fact that the majority of studies on resource competition between invasive and native species have focused on measuring biomass at one point in time or on final harvest data, and have not accounted for the dynamic nature of this process (see Trinder et al., 2013).

A potential source of bias in the interpretation of competition studies in invasion ecology is associated with the fact that several experiments have compared the competitive ability of invasive (dominant alien species) vs. that of native subordinate or transient species (*sensu* Grime, 1998). In this case, the selected native species would likely be negatively affected by resource competition with any dominant species, regardless of its native/alien status. This bias was evident in Vilà and Weiner's (2004) review of pair-wise competition experiments, which supported the general notion that invasive species are good competitors, although the authors warned that dominance by invasive species could depend on effects other than those associated with resource competition, including indirect competition, allelopathy (see Glossary; Weidenhamer et al., 1989; White et al., 2006), or phylogenetic and life form differences between species pairs.

The competitive ability of invasive vs. native species is dependent on the environmental conditions encountered in the introduced range (e.g., Alpert et al., 2000). Several authors have pointed out that evidence for a superior competitive ability of invasive species might be biased by the fact that the majority of studies have been conducted in highly productive environments (e.g., Kueffer et al., 2007), where invasive species tend to be better competitors than native species via a superior capacity to acquire resources more effectively than native species (e.g., Daehler, 2003; Matzek, 2012).

Finally, few studies have assessed the importance of resource competition relative to that of other mechanisms (e.g., Levine et al., 2003; Vilà and Weiner, 2004; White et al., 2006). In a review of 150 papers examining the impacts of alien plants, Levine et al. (2003) showed that fewer than 5% of those studies had confirmed the mechanisms responsible for the impact of alien plants (competition, allelopathy, or other processes), despite the majority having identified resource competition as a major mechanism underpinning their findings. The following provides is information on what is known about the competitive ability of invasive species vs. that of native species across a range of resource gradients, and on the factors that may hinder our capacity to assess the importance of resource competition in plant invasions.

### Competition for nutrients

Variations in the competitive ability of invasive species along resource gradients have received considerable attention (e.g., Grime, 1973, 1977, 2001; Newman, 1973; Tilman, 1982, 1988, 1997; Davis et al., 1998, 2000; Suding et al., 2004; Gross et al., 2005). A superior ability to acquire nutrients has been regarded as a major determinant of the successful establishment, spread, and persistence of invasive species, particularly in highly productive environments (e.g., Burke and Grime, 1996; Matzek, 2012), and several studies have shown that nutrient enrichment can be disproportionately more beneficial to invasive species than to natives (e.g., Huenneke et al., 1990; Witkowski, 1991; Milchunas and Lauenroth, 1995; Burke and Grime, 1996; Daehler, 2003; Lowe et al., 2003; Leishman and Thomson, 2005; Vinton and Goergen, 2006; Abraham et al., 2009; Sharma et al., 2010). Moreover, temporary increases in nutrient levels associated with natural or anthropogenic disturbances (see Glossary) may mitigate the negative effects of competition for nutrients (e.g., reduction in growth or lateral spread) with native species (Quinn et al., 2007), although the intensity of competition for nutrients may increase with increases in N availability (Mangla et al., 2011b). In contrast, decreases in nutrient levels may reverse the outcome of competition, with native species performing better than invasive species under low nutrient levels (e.g., Wedin and Tilman, 1993; Claassen and Marler, 1998).

Despite being regarded as better competitors for nutrients compared to native species in productive environments, many invasive species have also colonized unproductive environments (Groves et al., 2003; Funk, 2013). Increases in nutrient concentrations associated with natural or anthropogenic disturbances may promote plant invasions in these environments via a disproportionately beneficial effect on the competitive ability of invasive species over that of native ones, such as in serpentine ecosystems (O'Dell and Claassen, 2006) or in coastal dune communities (French, 2012).

In addition to possessing a superior capacity to acquire nutrients, many invasive species are known to reduce the level of nutrients available to co-occurring native species in invaded communities. For instance, Callaway and Aschehoug (2000) showed that the Eurasian forb, *Centaurea diffusa*, which is invasive in North America, had negative effects on nutrient (^32^P) uptake in North American bunchgrass species, likely due to a differential ability to use nutrients compared to native species. Suding et al. (2004) also showed that *C. diffusa* is better able to use P and is less limited by N compared to co-occurring native species in invaded communities, while, under low P, it appears to lose its competitive advantage and its response to resource competition is similar to that of native species.

### Competition for water

A superior capacity to compete for water may play a major role in promoting the establishment of alien species (e.g., Thebaud et al., 1996). In environments characterized by low water availability, native species are expected to be better competitors for water than alien species, due to a presumed adaptation to periodic water deficits. However, there is evidence showing that invasive species are better competitors even in environments characterized by low water availability (Nernberg and Dale, 1997; López-Rosas and Moreno-Casasola, 2012; Mason et al., 2012). For instance, Nernberg and Dale (1997) showed that the competitive ability of five native grasses was lower than that of the alien grass *Bromus inermis*, even under water stress. Mason et al. (2012) tested competition effects along gradients of water availability for a representative suite of species from coastal dune communities that had been invaded by *Chrysanthemoides monilifera* subsp. *rotundata* and showed that native species were often weak competitors compared to the invader, even under water stress, despite their adaptation to periodic water stress in native coastal environments, although native shrub species that are functionally similar to the invader were more effective at competing against the invader.

### Competition for light

Competition for light is generally regarded as an asymmetric type of competition (Yokozawa and Hara, 1992), which occurs when larger individuals obtain a disproportionate share of resources, relative to their initial size, suppressing the growth of smaller individuals (Begon, 1984; Weiner and Thomas, 1986; Keddy and Shipley, 1989; Weiner, 1990; Gerry and Wilson, 1995; Connolly and Wayne, 1996; Freckleton and Watkinson, 2001; see Glossary). Competition for light is considered a major determinant of the successful establishment of alien species, with many invaders outcompeting native species via a superior ability to capture light and via subsequent shading effects associated with a higher biomass production compared to natives (e.g., Hobbs and Mooney, 1986; Maule et al., 1995; Hutchinson and Vankat, 1997; Richardson et al., 2000; Morris et al., 2002; Kueffer et al., 2007; Iponga et al., 2008) and/or through related traits, such as a higher specific leaf area (e.g., Smith and Knapp, 2001; Iponga et al., 2008). For instance, Morris et al. (2002) showed that improved light capture and a greater stem elongation rate conferred the invasive shrub *Ligustrum sinense* with a competitive advantage over the native shrub *Forestiera ligustrina*. Such a superior competitive ability in light acquisition appeared to explain its higher photosynthetic capacity and resource use efficiency, as well as the observed fruit production of the invasive *L. sinense* vs. *F. ligustrina* (Morris et al., 2002). A superior capacity to compete for light compared to native dominant trees (*Acacia tortilis* and *Rhus lancea*) was reported to promote invasions by the alien tree *Schinus molle* in semi-arid savannas in South Africa (Iponga et al., 2008). Such a superior competitive ability was observed in alterations in canopy symmetry in native trees that were associated with a degree of intolerance to shading caused by the invader.

The formation of a large biomass by many invasive species is often associated with their superior capacity to compete for light and contributes to determining the magnitude of the impact of invasive species on native communities via shading effects (Grime, 2001). However, an invader's large canopy and/or biomass may be due to a superior capacity to compete below-ground for nutrients and water (e.g., Coomes and Grubb, 2000; Kueffer et al., 2007) rather than to a superior capacity to compete for light. Moreover, leaf dynamics or architecture may be more important than a large shoot biomass *per se* in conferring a high competitive ability (e.g., Grime, 2001). Assessments of competition for light should thus be examined in combination with assessments of the effects of competition for belowground resources.

### Competition for space

The allocation to vegetative vs. reproductive tissues is a function of the availability of underground space (McConnaughay and Bazzaz, 1991). Despite the fact that physical space is not a consumable resource (McConnaughay and Bazzaz, 1991; Bazzaz, 1996), its effects on the access to other resources such as water, nutrients, and light could play an important role in determining the outcomes of resource competition between alien and native species. The majority of studies referring to space constraints have examined patterns of invasions following disturbances creating gaps (increases in light availability) that can be colonized by ruderal invaders (e.g., D'Antonio and Vitousek, 1992; Hobbs and Huenneke, 1992; Thompson et al., 2001; Buckley et al., 2007), while the effects of space on competitive interactions between invasive and native species represent a major research need (Gao et al., 2014) that requires further investigation.

### The importance of phenology

Phenological differences resulting in early growth and in the initiation of significant size increases prior to those of native species may have a large impact on competitive interactions between invasive and native species (e.g., Tiley et al., 1996; Caffrey, 2001; Standish et al., 2001; Sala et al., 2007; Gioria and Osborne, 2010, 2013; Wilsey et al., 2011; Wolkovich and Cleland, 2011; Funk, 2013; Wolkovich et al., 2013). Early growth allows a species to exploit the available resources before other species and avoid competition for some resources during the initial stages of plant development. Thus, in the presence of phenological differences that allow an invasive species to grow earlier than native species, a high competitive ability in the invader may be less important or made unnecessary.

Drought avoidance is one particular example. In arid and semi-arid ecosystems, invasive species are not necessarily less drought-tolerant than native species (e.g., Williams and Black, 1994; Cleverly et al., 1997; Nernberg and Dale, 1997), and the successful establishment of some annual invaders is associated with a capacity to avoid drought stress (e.g., Solbrig, 1986; Fox, 1992) by completing their life cycles over the short period when water availability is high (see Alpert et al., 2000 and references therein). In some cases invasive species may possess a combination of water deficit evasion and tolerance mechanisms (Baruch and Fernandez, 1993). In a review of species traits of invasive species in low-resource environments, Funk (2013) showed that, in arid and semi-arid environments, three studies out of three showed that early germination was more pronounced in invasive rather than in native species under low water availability, indicative of potential phenotypic and/or adaptive responses to low water availability of invasive species resulting in phenological changes.

Phenological differences between invasive and native species represent a major confounding factor in determining the role of resource competition in the successful establishment of early growing alien species. Such differences should be accounted for as they allow an invasive species to avoid resource competition during the initial phase of development and confer an invader with competitive advantages (resource pre-emption) that are due to a capacity for early growth rather than to a superior competitive ability.

### Inter- and intra-specific competition

Competition at the early stages of plant development associated with small differences in initial size and growth between neighboring individuals may have long-term effects on competitive interactions (Weiner, 1993; Foster and Gross, 1997, 1998; Suding and Goldberg, 1999; Mangla et al., 2011b). Both interspecific competition between invasive and native species and intraspecific competition may thus affect the competitive ability of invasive and native species. To date, few studies have, however, examined the role of intra- and interspecific competition in determining the outcomes of competitive interactions between invasive and native species, and the results appear to be strongly species-specific.

For native species, interspecific competition with alien species appears to be the predominant form of competition (Lowe et al., 2003; Vasquez et al., 2008; Young and Mangold, 2008; Mangla et al., 2011b), although intraspecific competition may be important in determining the initial size of native seedlings, with potential effects on the outcome of competition with invasive seedlings (Mangla et al., 2011b). For some invasive species, intraspecific competition is often the predominant type of competition (Lowe et al., 2003; Vasquez et al., 2008; Young and Mangold, 2008; Blank, 2010; Mangla et al., 2011b; Skálová et al., 2013), likely reflecting stronger differences in competitive ability between invasive and native species than among individuals of the same species. For instance, examination of inter- and intraspecific competition among four native and invasive *Impatiens* species, Skálová et al. (2013) found that the invasive *I. parviflora* competed better in intra- vs. interspecific competition, while the invasive *I. glandulifera* performed better under interspecific competition with its congeners, although it may form a large aboveground biomass even in intraspecific competition experiments (Bottollier-Curtet et al., 2013).

The importance or intensity of intra- vs. interspecific competition may differ with the stage of the life cycle (e.g., Young and Mangold, 2008; Mangla et al., 2011b), since individual plants go through various physiological stages as they develop and competition occurs within and between stages for different individuals (Connell, 1983; Cameron et al., 2007; Mangla et al., 2011b). For instance, Mangla et al. (2011b) performed a range of competition experiments that tested the intensity and importance of intra- and inter-specific competition between two invasive annual grasses (*Bromus tectorum* and *Taeniatherum caput-medusae*), which are native to Eurasia and the Mediterranean region, and two native perennial grasses (*Pseudoroegneria spicata* and *Poa secunda*) that co-occur in their invasive range. They showed that native perennial grasses were subject to both intra- and interspecific competition with invasive annual species during early growth stages, but the type of competition differed among four harvests. This suggests that the relative importance of intra- vs. interspecific competition varies among harvests during the early stages of plant growth (Mangla et al., 2011b; see Trinder et al., 2013) and emphasizes the importance of examining competition at several points in time (Foster and Gross, 1997, 1998; Gibson et al., 1999), particularly when comparing species characterized by different life cycles (Gibson et al., 1999).

Bossdorf et al. (2004) warned that experiments aimed at identifying potential mechanisms leading to the successful establishment of invasive species may provide contrasting outcomes depending on whether the effects of intraspecific competition are accounted for or not, given that, under intense intraspecific competition, invasive populations may have lower fitness (van Kleunen and Schmid, 2003) and a reduced competitive ability (Bossdorf et al., 2004). Future studies should address this research gap, given the importance that intraspecific competition may play, particularly at the initial stages of invasion.

### Above- and below-ground competition

Plants use different parts (leaves vs. roots) to compete aboveground (for space and light) and belowground (for nutrients, water, and space) (Casper and Jackson, 1997; Schenk, 2006). The effects of belowground competition are not necessarily additive to those of aboveground competition (Wilson, 1988) but can be opposing and result in complex interactions (Wilson and Tilman, 1995). Roots of different species may interact so that those of one species may increase or decrease the concentration of different resources available to roots of other species (e.g., Schenk, 2006; Berger et al., 2008). It has been argued that, particularly in low productivity environments, belowground competition for nutrients is likely to be more important than aboveground competition for light in promoting the successful establishment and the persistence of invasive species (Dietz and Edwards, 2006). For instance, Kueffer et al. (2007) showed that belowground competition reduced significantly the growth of native juvenile trees in forests dominated by the invasive tree *Cinammomum verum*.

Despite the potentially different effects of above- and below-ground competition on the overall outcomes of resource competition between invasive and native species, the majority of studies in plant invasions have focused on observations of patterns in the aboveground vegetation and only few have examined belowground competition between invasive and native species (e.g., Gorchov and Trisel, 2003; Kueffer et al., 2007; Dehlin et al., 2008). How invasive and native plants compete above- and belowground for limiting resources, how they may alter the resources available to neighboring plants, and how they may alter the allocation of available resources to above- vs. belowground structures or to vegetative vs. reproductive structures in neighboring plants, have been seldom explored. More information is also required on how invasive species are associated with soil microbes, including symbiotic and associated N-fixing bacteria and mycorrhizae, in both high- and low-resource ecosystems (Funk, 2013). Mycorrhizae, in fact, may be important mediators of resource competition among plants (e.g., Hetrick et al., 1992; Bazzaz, 1996; Moora and Zobel, 1996), but information on this topic is scarce (Marler et al., 1999).

### Comparing alien dominant and native dominant species

Dominant species, regardless of their native/alien status, are considered to play a major role in regulating plant community dynamics (Grime, 1998; Smith et al., 2004), as they are responsible for most of the biomass in many communities, even where many transient or subordinate species are present (*sensu* Grime, 1998).

Comparisons of the competitive ability of alien vs. native dominants can be useful to assess the role of resource competition in plant invasions, at different stages of the invasion process. Few studies have however addressed this topic, and have done so mainly by comparing biomass as a measure of the competitive ability of a species, although differences in biomass might not be good indicators of a differential competitive ability given that dominant character of the species being compared. Among the studies addressing this question, Bottollier-Curtet et al. (2013) compared five dominant native species and five invasive species that co-occur along the Garonne River, France, and showed that, over a six-month-period, invasive dominants produced larger above- and belowground biomass compared to native dominants in 73% species pairs, suggesting a superior competitive ability of alien dominants over native dominants. Hovick et al. (2011) compared the competitive ability of two co-occurring dominant wetland species, the invasive *Lythrum salicaria* and the native *Typha latifolia*, by examining the colonizing success of seedlings of species other than the two dominants in monocultures of each dominant species. They found that *L. salicaria* reduced the success of colonizing species to a greater degree than *T. latifolia*, although differences in biomass explained little variation in colonizing success, and suggested that *T. latifolia* suppresses colonization via light reduction while *L. salicaria* does so via below-ground competition.

The potential role of dominance by native species in promoting the successful establishment of alien species has been recently emphasized (e.g., Smith et al., 2004; van Riper and Larson, 2009). In an experiment on a native Kansas grassland in which dominance by C_4_ grasses was manipulated (reduced by 25 and 50%), Smith et al. (2004) found that invasion by *Melilotus officinalis* was facilitated in plots dominated by dominant natives, due to their capacity to mitigate stressful environmental conditions, while reductions in dominance by C_4_ grasses reduced the establishment of the invader. These authors suggested that dominance is a key characteristic determining the establishment of alien species, depending on whether dominant native species exacerbate resource competition or mitigate stressful conditions (Smith et al., 2004). Similar findings for this species were reported by van Riper and Larson (2009), who showed that *M. officinalis* acted as a weak competitor and had no consistent effects on other species in a wheatgrass (*Pascopyrum smithii*) prairie, while, under sub-optimal conditions, it acted as a nursing plant, facilitating the growth of other species.

Since dominance represents an important plant community trait (Grime, 2001; Hovick et al., 2011), additional studies are needed to determine whether native dominants may facilitate or prevent plant invasions by alien dominants; whether differences in the competitive ability of alien and native dominants is key to the successful establishment of alien dominants and whether this competitive advantage is likely to be transient or long-lasting.

### Accounting for phylogenetic relatedness

Phylogenetic relatedness provides a measure of how much evolutionary history two species share and of their ecological similarity (Webb, 2000), with closely related species expected to have traits more similar than phylogenetically distant species, including traits involved in resource competition. Phylogenetic relatedness should thus be accounted for when one wants to capture differences in competitive ability among species, as this alone can explain part of the observed differences. This can be achieved by comparing phylogenetically related species, which allows minimizing trait differences among species associated with their evolutionary history (Powell and Knight, 2009).

Congeneric comparisons between phylogenetically related invasive and co-occurring native species thus represent an effective way of assessing the role of resource competition in the successful establishment of invasive species. Few studies, however, have examined resource competition between invasive and native congeners (Powell and Knight, 2009; Skálová et al., 2013).

Skálová et al. (2013) compared the effects of resource competition in four *Impatiens* species of different origin and invasive potential in central Europe: the native *I. noli-tangere*, and the aliens *I. glandulifera* (highly invasive), *I. parviflora* (less invasive) and *I. capensis* (potentially invasive). They found that *I. glandulifera* was the strongest competitor, followed by *I. parviflora*, particularly under low soil moisture conditions, while *I. capensis* was sometimes limited by alien competitors. These findings seem to indicate that a high competitive ability is important in determining the invasion success of *Impatiens* species and that invading congeners may outcompete the native *I. noli-tangere*. Powell and Knight (2009), in contrast, did not find any evidence for a superior competitive ability of invasive vs. native congeners. They compared the competitive ability of five *Cirsium* species co-occurring in northern California: the invasive *C. vulgare* and four native species, including the endemic *C. fontinale* var. *fontinale*. Contrary to their predictions, *C. fontinale* competed well even under high nutrient conditions and showed no significant reductions in biomass in competition experiments with *C. vulgare*, suggesting that its restriction to low-nutrient serpentine environments is due to factors other than a poor competitive ability in more productive habitats.

Congeneric comparisons between invasive and non-invasive alien congeners, conversely, can be useful to identify those traits that may confer invasive species with a high competitive ability. Several studies have shown that many invasive species possess higher values of competitively-related advantageous traits compared to non-invasive phylogenetically-related species (e.g., McDowell, 2002; Deng et al., 2004), including a higher N allocation to photosynthesis and N-use efficiency (e.g., Feng et al., 2007, 2008, 2009; Feng, 2008), and a higher specific leaf area (e.g., Grotkopp and Rejmánek, 2007; Feng et al., 2008; van Kleunen et al., 2010; Matzek, 2012), larger root biomass, and fast relative growth rate (e.g., Burns, 2004; Grotkopp and Rejmánek, 2007). These traits, however, are not necessarily good predictors of the successful establishment or persistence of an alien species (e.g., Leishman et al., 2010; Meisner et al., 2011), while studies examining resource competition between invasive vs. native congeners, under a range of environmental conditions, could provide important insights into the mechanisms underlying the successful establishment of invasive species.

### Resource competition and phenotypic plasticity

Phenotypic plasticity is the ability of a particular genotype to express a range of phenotypes in response to different environmental conditions (Bradshaw, 1965). High phenotypic plasticity in invasive plants has long been regarded as plant feature that may increase the probability of a species to become invasive (Baker, 1965; Pyšek and Richardson, 2006; Richards et al., 2006; Nicotra et al., 2010; Davidson and Nicotra, 2012). Phenotypic plasticity in functional traits may enhance niche breadth (Bradshaw, 1965; Sultan, 2001; Richards et al., 2006), i.e., the niche space or range of conditions required by a species, and may thus play an important role in the successful establishment of alien species in novel environments and its persistence in a community (Palacio-López and Gianoli, 2011).

A large number of studies have examined whether invasive species are more plastic than non-invasive or native species (e.g., Richards et al., 2006; Skálová et al., 2012), even in low resource environments (Funk, 2008), although contrasting results have been reported (Bossdorf et al., 2008; Davidson et al., 2011; Palacio-López and Gianoli, 2011; Matzek, 2012). Greater plasticity could indicate that (1) plasticity plays an important role in determining the successful establishment of alien species; and/or (2) plastic genotypes within species were selected during the invasion process (see Drenovsky et al., 2012 and references therein). In a recent meta-analysis of 75 phylogenetically related invasive/non-invasive species pairs, Davidson et al. (2011) found that invasive species had significantly higher phenotypic plasticity in a wide variety of morphological and physiological traits than non-invasive species, and they were nearly always more plastic in their response to increased nutrient availability than non-invasive species.

As described in Richards et al. (2006), phenotypic plasticity in an invasive species may be adaptive if it enables a genotype to (1) maintain fitness (fitness homeostasis) in unfavorable environments (“jack-of-all-trades” response to decreased resources), (2) increase fitness in favorable environments (“master-of-some” response to increased resources), or (3) both (“jack-and-master” strategy), i.e., a combination of both strategies, which corresponds to the “ideal weed” described by Baker (1965) and could allow a species to maintain high fitness across a broad environmental range (Mozdzer and Megonigal, 2012). The “master-of-some” strategy provides a mechanism by which higher plasticity of invasive species could enable invasive species to outcompete native species, thus facilitating the persistence of alien species in both low- and high-resource environments (Davidson and Nicotra, 2012; Mozdzer and Megonigal, 2012).

Recent findings show that high plasticity is not necessarily correlated to a higher fitness (e.g., Davidson et al., 2011; Matzek, 2012), and our knowledge of the effects of high plasticity as an important species trait in invasion processes is still limited (Hulme, 2008). Matzek (2012) tested the relative contribution of high trait values and high trait plasticity to relative growth rate (a proxy for fitness) for 10 closely related invasive and non-invasive *Pinus* species, and showed that in responding to higher N supply, superior trait values and not trait plasticity provides the better explanation for the performance of invasive species in a changing environment. Davidson et al. (2011) also showed that, despite invasive species having a higher phenotypic plasticity in 75 invasive and non-invasive species pairs, increases in resources did not result in higher fitness in invasive vs. non-invasive species comparisons.

Whether phenotypic plasticity resulting in higher fitness could be adaptive and/or indeed promote the successful establishment, spread, and long-term persistence of alien species has not been clarified (Daehler, 2003; van Kleunen and Fischer, 2005; Peacor et al., 2006; Richards et al., 2006; Davidson et al., 2011; Davidson and Nicotra, 2012; Matzek, 2012) and the effects of high phenotypic plasticity in both invasive and native plants on competitive interactions between invasive and native species requires further investigation.

### Role of rapid evolution in resource competition

There is evidence that invasive species may show a capacity to undergo rapid evolutionary changes associated with the novel environmental conditions encountered in the communities where they have become invasive (Thompson, 1998; Sakai et al., 2001; Lee, 2002; Stockwell et al., 2003; Bossdorf et al., 2005, but see e.g., Pahl et al., 2013). For some introduced species, adaptations to the novel conditions encountered in the introduced range resulting in an increased competitive ability may substantially alter competitive interactions between alien and native species over time (Bossdorf et al., 2005) and may play an important role in determining the persistence and the impact of an invader on native communities.

The release from natural enemies may alter competitive interactions via the increased reallocation of resources to reproduction and growth that were previously devoted to defense (e.g., Siemann and Rogers, 2001, 2003). This is the basis for the EICA hypothesis (Blossey and Nötzold, 1995; Willis et al., 2000; Vilà et al., 2003; Callaway and Ridenour, 2004), which has been used to explain why many invasive species often occur at greater densities and have a superior competitive ability in their invasive range compared to native species (e.g., Crawley, 1997; Keane and Crawley, 2002; Pergl et al., 2007).

There is little support for the “full” EICA hypothesis being a major factor in the successful establishment of alien plants (Thompson, 2014), with several studies finding no evidence of an increased performance of invasive species released from their specific herbivores, pathogens or parasites (Maron and Vilà, 2001; Thebaud and Simberloff, 2001; Bossdorf et al., 2004, 2005; Maron et al., 2004, 2014; Franks et al., 2008; Ridenour et al., 2008). An additional mechanism potentially leading to an increased competitive ability in invasive species was proposed by Feng et al. (2009), based on observations that the invasive shrub *Ageratina adenophora* had evolved an increased N allocation to photosynthesis (growth) and a reduced allocation to structural defenses (cell walls) in invasive populations compared to native populations. Moreover, if plants in invasive populations had more intra- than interspecific neighbors, they could evolve a reduced competitive ability (Evolutionary Reduced Competitive Ability; ERCA) that would allow the conservation of resources that would be otherwise required to compete against native species (Bossdorf et al., 2004). These resources could then be used for other processes that may lead to successful invasions, such as allelopathy (Prati and Bossdorf, 2004), developing plastic responses, or improving tolerance to herbivory (Bossdorf et al., 2004).

Not only may alien species respond to the novel conditions encountered in the introduced range, but also native species have the potential to adapt to the conditions created by the introduction of invasive species and evolve a capacity to compete with invasive species (Strauss et al., 2006b; Carroll et al., 2007; Mealor and Hild, 2007). Evolutionary changes leading to the genetic adaptation of invasive species and the co-evolution of invasive and native species may strongly affect resource competition between invasive and native species over time.

As changes in competitive ability may be evolutionary or due to phenotypic plasticity, understanding how resource competition between invasive and native species may change over time requires designing experiments that can identify which traits respond evolutionarily and which show a plastic response (and whether these responses will interact).

### A transient competitive advantage?

The temporal component of competitive interactions between invasive and native species is an important topic of research in invasion ecology. Increasing evidence shows that competitive advantages of invasive species over natives may be important only in the initial phases of the invasion process (e.g., Milchunas and Lauenroth, 1995; Claassen and Marler, 1998; Corbin and D'Antonio, 2004; Sala et al., 2007; Goldstein and Suding, 2014). Over time, the competitive ability of invasive species could decrease and ultimately result in the displacement of invasive species by natives (see Thompson, 2014 and references therein) through competitive exclusion (Corbin and D'Antonio, 2004; McGlone et al., 2012), although the dynamics of competitive interactions remain unclear.

Evidence for a superior competitive ability of invasive species over natives may thus have been biased by the design and relatively short-term duration of the majority of competition experiments involving native and alien species. Weiner (1993) emphasized the importance of the time scale in the study of competition, pointing out that the outcome of competition between two species may change over time. The major point is that short-term assessments may not give a good representation of the competitive ability of a species over the course of its development (see Milchunas and Lauenroth, 1995; Claassen and Marler, 1998; Corbin and D'Antonio, 2004; Sala et al., 2007; Goldstein and Suding, 2014, see Weiner, 1993; Trinder et al., 2013).

## Resource competition and invasibility

### Resource availability and other abiotic conditions

Resource competition is dependent on the spatial and temporal distribution of resources, and any change in the availability of limiting resources will inevitably alter the competitive balance between invasive and native species (Alpert et al., 2000). It has been shown that a highly heterogeneous distribution of resources may promote high species richness even in strongly competitive communities (e.g., Planty-Tabacchi et al., 1996; Stohlgren et al., 1999), via increasing niche differentiation, i.e., the use of different forms of a resource (Tilman, 2004; see Glossary).

An important question in invasion ecology is whether high-resource environments or environments characterized by low non-resource environmental stresses are more susceptible to invasions by alien plants compared to low-resource environments. Depending on the physiological amplitude of a species, the presence of a major abiotic stress may (1) prevent plant invasions regardless of the competitive abilities of native vs. alien species; (2) may prevent invasions only in combination with competition from native species; or (3) may slow an invasion but not prevent it (Alpert et al., 2000). If native species in a community were competitively superior over alien species, invasions would be prevented or slowed.

Vitousek et al. (1997) suggested that only few ecosystems are unlikely to be invaded by alien species. Despite difficulties in making robust generalizations on the characteristics of invaded communities (Rejmánek et al., 2005), there is evidence that plant communities differ in their degree of invasibility, i.e., their vulnerability to invasions (Lonsdale, 1999; Davis et al., 2000, 2005; see Glossary). High resource environments and/or environments characterized by low abiotic stresses indeed appear to be more invasible than low-resource environments (e.g., Huenneke et al., 1990; Burke and Grime, 1996; Daehler, 2003; Gross et al., 2005; Stohlgren et al., 2008; Moles et al., 2012; see Funk and Vitousek, 2007; Funk, 2013), although we have already mentioned a potential bias associated with a larger number of studies in high- vs. low-resource environments (e.g., Kueffer et al., 2007; Funk, 2013).

A lower invasibility in low-resource environments is often attributed to the assumption that native species should possess a competitive advantage over invasive species associated with their being adapted to the growth-limiting conditions characterizing such environments, while alien species have not had the opportunity to adapt to the local environmental conditions at the time of introduction (“the paradox of invasion,” Alpert et al., 2000; Sax and Brown, 2000; Daehler, 2003). However, jack-of-all-trade alien species can perform as well as natives under a broad range of environmental conditions, thus high resources or low environmental stresses are not good predictors of successful invasions. Many alien species have, in fact, invaded low-resource environments (Funk and Vitousek, 2007; Funk, 2013), including arid and semi-arid grasslands (Fowler, 1986), serpentines (e.g., Huenneke et al., 1990; O'Dell and Claassen, 2006; Vallano et al., 2012), or coastal dunes (e.g., French, 2012; Gioria and Osborne, 2013). Moreover, low nutrient availability may not affect competition (Kolb and Alpert, 2003) and, in one instance, low resources have been found to even promote invasions (Cleverly et al., 1997; see Funk, 2013).

This apparent paradox has been explained with some of the theories described in this paper, including high phenotypic plasticity, a capacity for rapid evolutionary adaptive changes, the release from enemies, high environmental heterogeneity, or a superior competitive ability characterizing invasive species that are native to species-rich regions where resource competition is high (Sax and Brown, 2000). Moreover, in low-resource environments, any temporary increase in available resources can be disproportionally beneficial to invasive plants (e.g., Hobbs and Mooney, 1991; Dukes and Mooney, 1999; Kolb et al., 2002; Thomsen et al., 2006; Abraham et al., 2009), although the temporal dimension of these effects requires additional investigations.

### Resource acquisition vs. resource conservation traits

A question that has received increasing attention in invasion ecology is whether invasive species possess more resource acquisition or resource conservation traits, in high vs. low resource environments (Crawley et al., 1996; Funk and Vitousek, 2007; Tecco et al., 2010; Funk, 2013). A superior competitive ability of alien species over that of natives is often associated with a high ability to acquire and retain resources (Tecco et al., 2010), although the traits associated with this ability are strongly habitat-dependent (Theoharides and Dukes, 2007). Successful invaders tend to possess traits associated with rapid resource acquisition and growth, including nutrient-rich leaves, with a high specific leaf area, and a short lifespan, in high resource environments (Burns, 2004, 2006; Blumenthal, 2005; Leishman and Thomson, 2005; Rejmánek et al., 2005; Grotkopp and Rejmánek, 2007; Leishman et al., 2007; Feng, 2008; Feng et al., 2008; van Kleunen et al., 2010; Matzek, 2012), while in low-resource environments, invasive species vary significantly in their strategies to cope with low resource availability, possessing either traits indicative of resource conservation or resource acquisition strategies (Funk, 2013). In particular, invasive species appear to use nutrients more efficiently than co-occurring native species in low-nutrient soils, while in light-limited systems, invasive and native species are similar in their water use efficiency (Funk, 2013).

The life form of the invaders may affect the results of studies addressing this research question. In an investigation comparing functional traits in native and alien species of central-western Argentina, across contrasting ecosystem types and management regimes, Tecco et al. (2010) showed that woody alien species possessed significantly more acquisitive sets of attributes than native species, while they did not detect any significant difference in trait syndrome (acquisitive vs. conservative) between herbaceous alien and native species.

It is worth noting that acquisitive vs. conservative strategies or syndromes in low-resource ecosystems are not necessarily incompatible, and that enhanced resource acquisition and the sparing use of those resources in biomass production could arguably go hand in hand, and differences with natives could be dependent upon the “opportunistic” response with a higher capacity of invasive species to exploit pulses or enhanced resource levels being important (see also Grime and Hunt, 1975 on variation in relative growth rate). Future research should examine the extent to which differences in functional strategies (acquisitive vs. conservative) in invasive and native species may help predict the outcomes of competitive interactions between invasive and native species.

## Disentangling resource competition from other mechanisms

### Competition, recruitment limitation

Recruitment limitation in both alien and native species may affect the outcomes of resource competition and resource competition dynamics (e.g., Hamilton et al., 1999; French et al., 2011; Gioria et al., 2012). In his neutral theory of ecological equivalence of species in a community, Hubbell (2001, 2006) proposed that recruitment limitation can delay competitive exclusion associated with asymmetric competition. In a theoretical study on plant competition for space, Hurtt and Pacala (1995) showed that competitively inferior species can coexist with dominant, competitively superior species, when the dominant species is recruitment limited. Thus, in the absence of niche differentiation (see Glossary), the outcomes of competition should mainly depend on differences in the competitive ability of alien vs. native species, with competitively superior species ultimately excluding competitively inferior species. Recruitment limitation in invasive species that are better competitors than natives should thus delay competitive exclusion of natives, while recruitment limitation in native species should exacerbate the effects of competition with competitively superior invasive species. This is consistent with the results of experimental studies showing that recruitment limitation in native species exacerbated the competitive effects of invasive species (Tilman, 1997; MacDougall, 2004), while Seabloom et al. (2003) showed that dominance patterns by alien annuals were likely caused by recruitment limitation of native perennial species rather than by a superior competitive ability of alien species in a California grassland community.

The importance of recruitment limitation relative to that of resource competition in determining the successful establishment of alien species and in the persistence of invasive species in a community deserves further investigations, as recruitment limitation in native species may increase the invasibility of native communities regardless of the competitive ability of the introduced alien species (Hamilton et al., 1999; Turnbull et al., 2000; French et al., 2011; Gioria et al., 2012).

### Niche vs. fitness differences

Niche differences reflect differences in resource use or response deriving from long-term competitive interactions among the species present in a community (Bazzaz, 1996; see Glossary). In contrast, fitness differences reflect differences in competitive ability (e.g., Tilman, 1988), in fecundity, or in the susceptibility to predators and pathogens (see MacDougall et al., 2009), and can be estimated by differences in growth rate (Adler et al., 2007). Niche and fitness differences have opposite effects on the outcomes of competition (Chesson, 2000) and may strongly affect the importance of resource competition in the successful establishment, spread, and persistence of alien species, although their importance relative to that of resource competition requires further investigations.

MacDougall et al. (2009) proposed an interesting framework to unify previous theories on coexistence between alien and native species along a fitness and niche differences axis. Niche differences between alien and native species may facilitate the establishment of alien species (MacDougall et al., 2009), by allowing an alien species to avoid resource competition, and may favor coexistence (Adler et al., 2007). In contrast, in the absence of niche differences, fitness differences will lead to the competitive exclusion of species with a comparatively low average fitness. This framework also encompasses the Empty Niche Hypothesis (e.g., Stachowicz and Tilman, 2005), which postulates that the presence of empty niches, i.e., niches not occupied by any native species, may promote invasions by niche-differentiated alien species due to the incomplete use of resources by native species. Thus, even if an alien species was a poor competitor, it could establish and, ultimately, become invasive in the presence of empty niches.

### Indirect competition

Besides competition for resources, other types of interactions (e.g., indirect competition and allelopathy) may affect the establishment, spread, and persistence of alien species in invaded communities. Indirect competition includes competition for shared pollinators and apparent competition (see White et al., 2006). Competition for shared pollinators often results in a reduced visitation of pollinators to native species associated with the presence of an alien species (e.g., Brown et al., 2002; Moragues and Traveset, 2005; Munoz and Cavieres, 2008; Kandori et al., 2009; Morales and Traveset, 2009; Palladini and Maron, 2013) and may be exacerbated by the dominance of invasive species in a community (Bjerknes et al., 2007; Morales and Traveset, 2009). Despite an increasing interest in this type of competition, studies assessing the importance of resource competition relative to that of competition for shared pollinators are scarce. Among these studies, Palladini and Maron (2013) showed that, although the invasive perennial forb *Euphorbia esula* reduced substantially pollination visitation to native annual *Clarkia pulchella*, native plants were not pollen-limited, suggesting that resource competition was more important than apparent competition in conferring *E. esula* a competitive advantage over *C. pulchella*.

Apparent competition between plants occurs when one species alters the abundance or the distribution of consumers and thus the consumption of other species (Holt, 1977; Holt and Kotler, 1987; Connell, 1990). More specifically, apparent competition may occur (1) when a species provides a consumer with a non-food resource, e.g., shelter, allowing the consumer population to increase and spread, with subsequent negative effects on the native species, or (2) when both plant species provide a food-resource to a food-limited consumer (e.g., Sessions and Kelly, 2002; Orrock et al., 2008; Dangremond et al., 2010; Recart et al., 2013).

Relatively few studies have examined the role of apparent competition in promoting plant invasions, although the interest in this type of competition has increased. There is evidence that apparent competition between alien and native species have significant negative consequences for native species (Sessions and Kelly, 2002; Orrock et al., 2008; Dangremond et al., 2010; see White et al., 2006 for a review), although its effects appear to be strongly context-dependent (e.g., Orrock and Witter, 2010; Mattos et al., 2013; Recart et al., 2013). Such negative effects of apparent competition could contribute to reduce the competitive ability of native species over that of invasive species. The presence of alien species may, however, have positive effects on the competitive ability of native species by reducing the pressure of generalist herbivores on native species. Recent studies (Jacquemart et al., 2013) and meta-analyses (Parker and Hay, 2005; Parker et al., 2006) indicate that some generalists have a preference for alien plant hosts over native plants, while some alien plants may negatively impact on the survival of generalist herbivores (Tallamy et al., 2010), and may thus benefit native species, altering the competitive balance between alien and native species. How apparent competition may affect or interact with resource competition in determining the establishment of alien species remains unclear and requires additional investigations.

### Allelopathy

Allelopathy can be defined as the effect of one individual on its neighbors associated with the release of chemical compounds from roots, shoots, leaves, or flowers (Rice, 1984, see Glossary). The Novel Weapon Hypothesis postulates that the invasiveness of certain alien species could depend on their ability to produce secondary metabolites that are evolutionarily novel in their introduced range and that interfere with native plants, microbes, pathogens, or generalist herbivores and reduce the growth of native plants (e.g., Callaway and Aschehoug, 2000; Bais et al., 2003; Hierro and Callaway, 2003; Callaway and Ridenour, 2004; Callaway et al., 2004; Pisula and Meiners, 2010; Uddin et al., 2014). The production of allelochemicals generally has effects that are greater in a species' introduced range than in its native range (e.g., Callaway and Aschehoug, 2000; Bais et al., 2003; Hierro and Callaway, 2003; Callaway and Ridenour, 2004; Callaway et al., 2004, 2008; Prati and Bossdorf, 2004; see Inderjit et al., 2011 and references therein for a discussion on evolutionary changes in allelochemical effects).

While a capacity to produce chemical defenses against competitors has been viewed as a factor potentially conferring a species with a competitive advantage over neighboring species, recent evidence shows that increases in the production of allelochemicals in response to intense resource competition may substantially reduce the growth of the same species and increase their palatability to herbivores, with potential negative effects on their ability to compete for resources (Rasher and Hay, 2014). The full ecological implications of allelopathy on resource competition between alien and native species remain unclear. Future research on this topic must, however, be reconciled with the well-known difficulties associated with separating the effects of resource competition from those of allelopathy in natural systems (see Inderjit and del Moral, 1997).

## Future research directions

### Global change and resource availability

Global environmental changes, such as climate change, increasing atmospheric CO_2_, and atmospheric N deposition, will affect the spatio-temporal distribution and dynamics of the resources available to plants (Wedin and Tilman, 1996; Dukes, 2000, 2002b; Smith et al., 2000). Such changes will inevitably affect resource competition between alien and native species (e.g., Vitousek, 1994; Vitousek et al., 1996; Dukes and Mooney, 1999; Dukes, 2000, 2002b; Novoplansky and Goldberg, 2001; James et al., 2006; Bradley et al., 2009; Firn et al., 2010).

Competition experiments can provide important insights into the effects of global environmental changes on resource competition between alien and native species, although, to date, expected changes in competitive interactions among species have been mainly based on individual species responses (see Goldstein and Suding, 2014 and references therein). An interesting study on the effects of climate change on competitive interactions between alien and native species is that of Goldstein and Suding (2014), who examined changes in resource competition between alien grasses and California coastal sage scrub species associated with projected changes in rainfall patterns in additive competition experiments, under three rainfall treatments: (1) frequent small events, (2) infrequent large events, and (3) infrequent small events. Rainfall amount and frequency altered competitive interactions between California coastal sage scrub and grasses. In the first year, the competitive effect of annual grasses on shrub seedlings was strongest under treatment (1), while in the second year, the established shrubs started exerting strong competitive effects on grasses, particularly under treatment (3) with a low total rainfall. These findings suggest that reductions in both rainfall frequency and total rainfall may alter plant community composition and invasion dynamics via alterations in competitive interactions between alien grasses and native species.

Increasing levels of N deposition associated with anthropogenic activities are expected to favor the establishment and long-term persistence of invasive species (e.g., Dukes and Mooney, 1999; Bobbink et al., 2010; Vallano et al., 2012). The strong positive growth and competitive response of many invaders to N addition (e.g., Huenneke et al., 1990; Witkowski, 1991; Milchunas and Lauenroth, 1995; Burke and Grime, 1996; Daehler, 2003; Lowe et al., 2003; Leishman and Thomson, 2005) suggest that N deposition may increase the competitive ability of invasive species vs. that of natives, particularly in low-nutrient environments, where native species are adapted to nutrient-deficient soils (e.g., Huenneke et al., 1990; Burke and Grime, 1996; Kolb et al., 2002; Lowe et al., 2003; Thomsen et al., 2006; Vallano et al., 2012).

Increasing concentrations of atmospheric CO_2_ are also expected to impact on resource competition between alien and native species. Evidence shows that elevated CO_2_ stimulates photosynthetic carbon gain and net primary production, improves nitrogen use efficiency, and decreases water use, thus removing some moisture constraints (e.g., Ainsworth and Rogers, 2007; Leakey et al., 2009). In competition-free systems (experiments conducted using monocultures), invasive plants seem to respond strongly to increases in CO_2_, in the short-term, but their response in competitive systems could be reduced (Bazzaz and McConnaughay, 1992; Dukes, 2000). How projected global environmental changes may affect resource competition between alien and native species in competitive systems still remains unclear and deserves further investigation (Dukes, 2002a; Vallano et al., 2012; Goldstein and Suding, 2014).

### Alterations in resource availability by established alien species and secondary invasions

There is strong evidence that many invasive species are capable of altering the levels of available resources in invaded ecosystem (e.g., Vitousek et al., 1996; Lindsay and French, 2004, 2005; Ehrenfeld, 2010; Vilà et al., 2011; Pyšek et al., 2012). Plant invasions may do so by altering the composition of native communities and patterns of dominance among plant functional types, including herbaceous vs. woody plants, C_3_ vs. C_4_ species, or nitrogen-fixing vs. non nitrogen-fixing species. These changes can strongly affect the distribution and dynamics of soil nutrients and other resources (e.g., Vitousek et al., 1987; Fogarty and Facelli, 1999; Gill and Burke, 1999; Ehrenfeld, 2010), by alterations in nutrient availability associated with the introduction of nitrogen-fixing invaders (Vitousek and Walker, 1989; Gioria et al., 2011), increased light availability via a reduction in the biomass of resident species (Flory and Bauer, 2014), or reductions in the amount of available water by deeply-rooted invaders, such as salt cedar *Tamarix* (Vitousek and Walker, 1989).

Invasive species may also alter the soil biota through plant-soil feedbacks (e.g., Kulmatiski et al., 2008; Suding et al., 2013), with potential negative effects on native species, such as those caused by the introduction of soil-borne pathogens, herbivores, or parasites, or positive effects, such as those associated with increases in mycorrhizal fungi or nitrogen fixing bacteria (Klironomos, 2002; Callaway et al., 2004, 2008; Ehrenfeld, 2010; Gioria and Osborne, 2013). In contrast, plant-soil-feedbacks may be beneficial to the invader itself (e.g., Reinhart and Callaway, 2006; Bever et al., 2010; Smith and Reynolds, 2012; but see Levine et al., 2006; Shannon et al., 2012; Suding et al., 2013). Shannon et al. (2012) showed that plant-soil feedbacks may change with modifications in competitive interactions between invasive and native species, as an invasion process progresses.

Changes in the availability of resources associated with plant invasions may thus create conditions that may either increase or decrease the competitive ability of invasive species vs. that of native or other alien species. How these changes will affect resource competition between alien and native species is a key to improving our understanding of the long-term implications of plant invasions on native communities. Moreover, such changes could create conditions that facilitate secondary invasions, i.e., the establishment of other alien species in a community (e.g., Gioria et al., 2011). The study of secondary invasions could provide important insights into competitive interactions among alien dominant species and resource competition dynamics, and represents an important topic that has so far received little attention.

### Issues and guidelines for future studies

Future research studies must address a number of deficiencies that have characterized many competition experiments in plant invasion ecology and that might have hindered our capacity to predict the role of resource competition in plant invasions. Throughout this review, we have pointed out a number of research needs on the role of resource competition in plant invasions, including that for more direct measurements of the competitive ability of invasive and native species (see also Trinder et al., 2013), under a broad set of environmental conditions representative of those that may be encountered in the field, as well as testing the interactive effects of multiple abiotic conditions (see Nernberg and Dale, 1997; Sammul et al., 2000), given that plants are typically subjected to more than one environmental source of stress and that the response to multiple stress factors may not be predictable on the basis of each applied individually (Mittler, 2006).

Another issue characterizing competition studies on plant invasions is that many pair-wise competition experiments have compared the competitive ability of alien and native species possessing contrasting life forms, such as annuals vs. perennials (Claassen and Marler, 1998; Groves et al., 2003; Abraham et al., 2009; Mangla et al., 2011a), forbs vs. grasses (Callaway and Aschehoug, 2000; Sharma et al., 2010), or herbaceous vs. woody species (Eliason and Allen, 1997; Bottollier-Curtet et al., 2013), with potential significant effects on the outcomes of competitive interactions. Clearly, different life forms may be associated with variations in the timing and magnitude of resource use, regardless of their native/alien status. For instance, the dominance and suppression of winter and summer annuals does not depend on differences in the competitive abilities among these life forms, but mainly on differences in the timing of soil disturbance (Bazzaz, 1996). Similar considerations pertain to comparisons of the competitive ability of annual vs. perennial species or that of herbaceous vs. woody species. Future experiments should thus account for differences in the type and timing of resources required by different life forms for both alien and native species, since competition experiments where different life forms are sown synchronously might fail to represent realistic competition dynamics in the field and possibly overestimate the effects of resource competition and the competitive ability of invasive species.

Resource competition is a dynamic process (Trinder et al., 2013) and is strongly linked to resource availability dynamics. Competitive interactions between invasive and native species should thus be examined over time and should be carried out at different stages of development, for both invasive and native species (e.g., Weiner, 1993; Mangla et al., 2011b; Trinder et al., 2013). It has been suggested that, after an initial phase where pre-adapted species become dominant, during a second phase, they can then spread into low resource environments due to shifts in life history traits either via plastic responses or adaptive evolution or both (Dietz and Edwards, 2006). Future research in plant invasions should thus examine how resource competition may change over time due to phenotypic plasticity and/or the ability of alien species to evolve and adapt to the new conditions experienced in their introduced range, during each phase of the invasion process, accounting for the temporal scale of these processes.

Interpreting the outcomes of resource competition strongly depends on the way competition is measured (Goldberg et al., 1999; Weigelt and Jolliffe, 2003; Freckleton et al., 2009). While competition intensity, i.e., the absolute magnitude of competitive effects (see Glossary; Grace, 1991, 1995) has been examined extensively, the importance of competition, i.e., the effects of resource competition on community composition or community dynamics relative to those of other types of interaction (see Glossary; e.g., Goldberg, 1994), has been assessed less frequently. While intensity refers mainly to the present process of competition, its importance also reflects the results of past competition (Welden and Slauson, 1986). Assessing the role of resource competition in plant invasions requires information on both its intensity and its importance relative to that of other mechanisms or processes.

**Figure 1:**
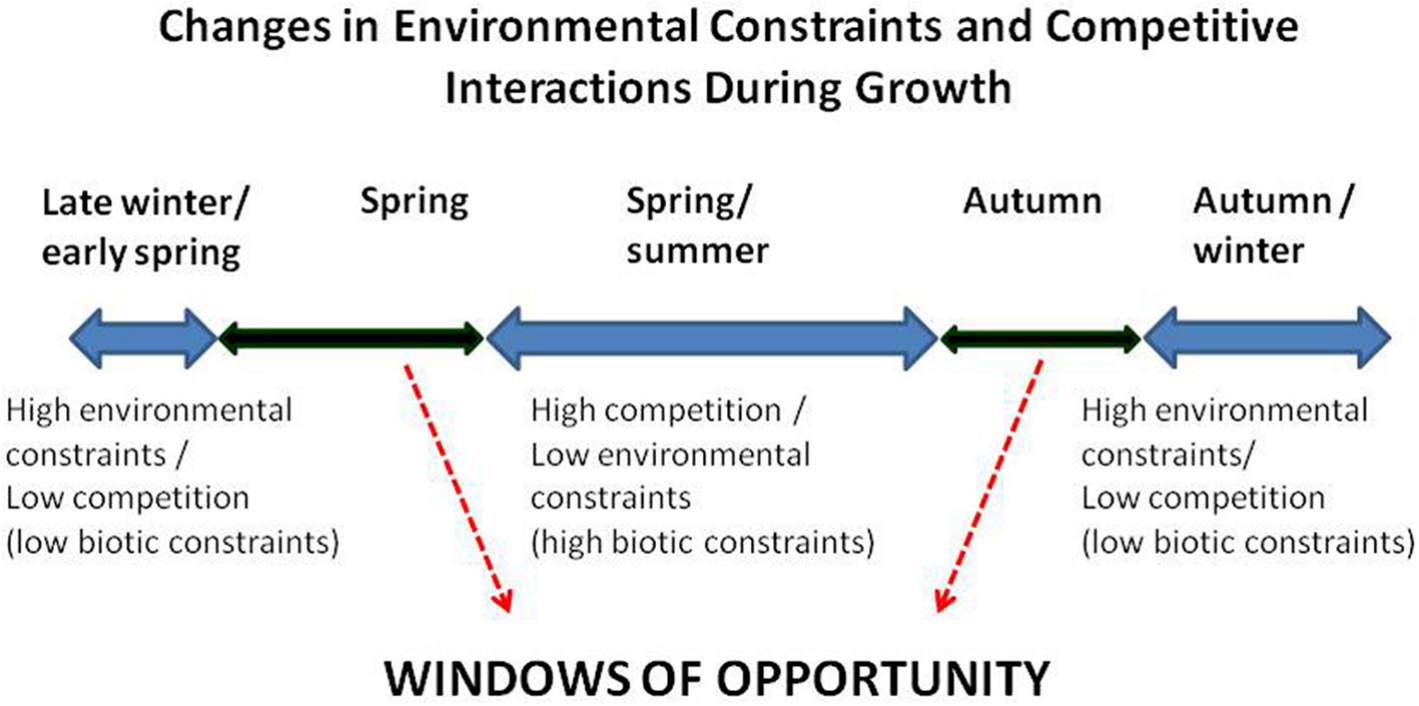
**Diagrammatic representation of changes in environmental constraints and competitive interactions during vegetative growth and development over the course of a year**. Note that in spring and, to some extent, in autumn, competition will be low, although there could be high to moderate environmental constraints to growth and development that could impact on any introduced alien plant species. At other times, competitive interactions will be high, with the possibility of biotic constraints associated with near optimal environmental conditions during the main growth period. The “windows of opportunity”, designated by black arrows, represents periods of reduced competition, with the spring “window” likely to be greater than the autumn “window”, particularly in cases with low vegetation cover or where the onset of growth-limiting environmental constraints occur rapidly. Establishment in the autumn “window” will be severely constrained by gradually decreasing temperatures and day length. The maintenance of a long-lived canopy well into the autumn “window”, a feature of many invasive plant species, will also reduce recruitment or end-of-season growth and seed germination of native species. Even if some growth or seed germination does occur, these individuals are unlikely to overwinter.

Phylogenetic and niche differences between alien and native species may confound the effects of resource competition and should be accounted for when predicting the outcomes of resource competition among species. Phenological differences between alien and native species should also be accounted for when examining the role of resource competition in plant invasions, as they may strongly affect competitive interactions, particularly at the early stages of plant development, and could explain why some invasive species may not be strong competitors if they can take advantage of temporal windows of opportunity when competitive interactions are weak or non-existent (Figure 1).

Coevolutionary responses among competing plants have been generally neglected (Leger and Espeland, 2010; Lankau, 2012). However, assessments of the reciprocal evolutionary responses of invasive and alien species after the introduction of an invasive species could improve substantially our capacity to predict resource competition dynamics (Strauss et al., 2006a; Mealor and Hild, 2007) and could provide some insights into why some initially successfully invaded locations are subsequently replaced by native or other alien species, as has been observed in some instances (Gioria et al., 2011; Thompson, 2014).

Future studies should also examine more extensively how clonal integration, i.e., resource sharing among interconnected ramets, or plant parts (see Glossary; e.g., Alpert and Mooney, 1986; Alpert, 1996; Liu et al., 2006; Xu et al., 2012), could affect the competitive ability of clonal invaders. Clonal integration may buffer the effects of the heterogeneous distribution of soil resources (Hutchings and Wijesinghe, 1997) and has been shown to affect the response of invasive clonal species to variations in light (Xu et al., 2012), water (You et al., 2013), and nutrient availability (You et al., 2014). Despite several invasive species being clonal, the role of clonal integration on competitive interactions for resources has been examined only recently (Wang et al., 2008; You et al., 2013), with studies showing that it can promote invasiveness under heterogeneous conditions (You et al., 2013, 2014) via its effects on growth, biomass allocation, and photosynthetic efficiency (Wang et al., 2008; Xu et al., 2012; You et al., 2013) and facilitate colonization under competitive situations (Xiao et al., 2011).

It is beyond the scope of our review to propose a detailed sampling framework or to describe in detail the drawbacks and advantages of experimental designs for assessing the importance and intensity of resource competition between alien and native species (see Trinder et al., 2013 for a discussion of the issues characterizing assessments of resource competition among plants). More complex experiments, over temporal scales that allow assessing competition dynamics, are needed to improve our capacity to characterize the role of resource competition in plant invasions, as well as to predict the long-term implications of the introduction of alien species. A common sampling protocol to assess competition dynamics and compare the competitive ability of alien vs. native species, based on standardized measures of competition importance, competition intensity, competitive ability, or competition effects (e.g., Grace, 1995; Goldberg et al., 1999; Brooker and Kikividze, 2008; Freckleton et al., 2009; Damgaard and Fayolle, 2010), would allow comparisons the results of multiple studies in multiple regions and ecosystem types, including comparisons of the competitive ability of selected invasive species, across ecosystem types and geographical regions, thus providing insights into the effects of phenotypic plasticity and evolutionary changes on the importance of resource competition in plant invasions.

## Conclusions

Resource competition has long been regarded as a major determinant of the successful establishment and spread of alien species and their long-term persistence in invaded communities, although its importance relative to that of other mechanisms remains unclear. As resource competition is a dynamics process, its role in plant invasions will inevitably change over time, not only due to changes in available resources associated with disturbances, global environmental changes, or changes promoted by the invaders themselves, but also due to plastic responses and evolutionary changes that may occur during invasion processes in both invasive and native species. In this review, we highlighted the most pressing research needs in this field and described a range of factors that may confound our capacity to determine the importance of resource competition in plant invasions, including phenological differences resulting in competition avoidance, niche and fitness differences, phylogenetic relatedness, recruitment limitation, indirect competition, or allelopathy. Improving our understanding of the role of resource competition in plant invasions and its dynamics does not only represent a key ecological question but is essential to predicting the long-term impacts of plant invasions and of how they may interact with other global environmental changes.

### Conflict of interest statement

The authors declare that the research was conducted in the absence of any commercial or financial relationships that could be construed as a potential conflict of interest.

## Glossary

*Allelopathy*: the negative effects of one individual on its neighbors associated with the release of chemical compounds from roots, shoots, leaves, or flowers (Rice, 1984).
*Apparent competition*: an indirect type of competitive interaction that occurs when one species alters the abundance or the distribution of consumers and thus the consumption of other species (Holt, 1977). Apparent competition may occur when a species provides a consumer with a non-food resource, e.g., shelter, allowing the consumer population to increase and spread, with subsequent negative effects on the native species, or when both plant species provide a food-resource to a food-limited consumer (Dangremond et al., 2010).
*Asymmetric competition*: an unequal division of resources among competing plants (Freckleton and Watkinson, 2001). It occurs where some individuals or some species remove a disproportionately large amount of resources (Freckleton and Watkinson, 2001). Where asymmetric competition occurs due to differences in size that confer an initial size advantage, the competitive effect is larger than the difference in size, meaning that if an individual is twice the size of another individual, the competitive effect must be more than twice or the larger individual take up more than twice the resources available (Weiner, 1993).
*Clonal integration*: resource sharing among interconnected ramets (Alpert and Mooney, 1986).
*Competition importance*: the relative impact of resource competition, among other processes, on plant fitness, community composition or population dynamics (Welden and Slauson, 1986). Competition importance is an ecological concept.
*Competition intensity*: the degree to which resource competition by neighboring individuals reduces the performance of an individual (or species) below a value when no neighbors are present (Welden and Slauson, 1986). Competition intensity is a physiological concept.
*Competitive ability*: the ability of a species to acquire limiting resources and/or a capacity to cope with low resource levels or to reduce the availability of resources to its neighbors. The competitive ability of a species has two components: (1) **competitive effect**: the ability to take up resources and thereby reduce the amounts available for other plants (Goldberg, 1990); (2) **competitive response**: the ability to perform well even though resource levels are reduced by the competitors (Goldberg, 1990).
*Disturbance*: the partial or total destruction of the plant biomass that can arise from the activities of herbivores, pathogens and humans (trampling, mowing, and plowing), and from phenomena such as wind damage, frosts, droughts, soil erosion, and fire (Grime, 2001).
*Indirect competition*: complex competitive interactions involving more than two species, resulting from the effects of one species on a third species via effects on a second species (e.g., White et al., 2006).
*Invasive alien species*: a subset of **alien** species, i.e., species that have been introduced either intentionally or unintentionally outside their native geographical range, which have become **naturalized** plants that produce reproductive offspring, i.e., have formed self-sustaining populations without direct human intervention, and have become invasive, i.e., are found often in very large numbers, at considerable distance from the parent plants, thus having the potential to spread over a large area (Richardson et al., 2000). Approximate scales: >100 m in <50 years for species spreading by seeds and other propagules (for dioecious taxa that rely exclusively on seeds for reproduction, this applies only after the introduction of both sexes); >6 m in 3 years for species spreading by roots, rhizomes, stolons, or creeping stems (Richardson et al., 2000).
*Invasibility*: the susceptibility of a community to the colonization and establishment of introduced alien species (Lonsdale, 1999). Invasibility can be quantified as the probability of successful establishment per arriving propagule (Davis et al., 2005). Invasibility describes a community's potential to be colonized, while the realization of that potential is dependent on the presence and abundance of propagules (Davis et al., 2005).
*Niche differentiation*: differential resource use or response resulting from long-term competitive interactions between species in a community (Bazzaz, 1996).
*Resource competition*: a negative interaction between individuals or species associated with a requirement for shared limiting resources resulting in a reduction in one or more fitness components at the individual level or at the population level (Goldberg et al., 1999). From a functional point of view, competition can be regarded as an alteration of the processes of (1) “acquisition” of resources, (2) their “allocation” to different parts, and (3) the “deployment” of these parts in space (Bazzaz, 1996), by neighboring individuals.
*Resource*: consumable or depletable “supply factors” that are required by plants for maintenance, growth, and reproduction (e.g., Harper, 1977), including light, water, nutrients, oxygen, and CO_2_.
*Non-resource condition*: include “non-consumable” factors, such as temperature. Some factors, such as light, can be both resource and non-resource conditions (Bazzaz, 1996).
*Stress*: physical, chemical, and biological constraints that restrict photosynthetic production. These include shortage of light, water and mineral nutrients, or suboptimal temperatures (Grime, 2001).
